# Demographic Acceleration, Evolving Disease Burdens and Shortfalls in Provision–the Future of Older Adult Psychiatry

**DOI:** 10.1192/bjo.2026.11346

**Published:** 2026-06-30

**Authors:** James Anslow, Theodoros Pashalis, Jennifer Parker, Donncha Mulin

**Affiliations:** 1 South London and Maudsley, London, United Kingdom; 2 Cambridgeshire and Peterborough, Cambridge, United Kingdom; 3 Avon and Wiltshire, Bristol, United Kingdom; 4 Royal Edinburgh Hospital, Edinburgh, United Kingdom

## Abstract

**Aims:**

There is a significant and rapidly widening chasm between the demand for Old Age Psychiatry and what the current workforce can deliver.

We aimed to review the changing burden of psychiatric disease in the 55–65 age group to help predict future needs, the current workforce providing this care and the training providing this in the future.

**Methods:**

We have collected data regarding the rate of complex psychiatric diagnoses in 55–65 and 65 and older people in South London and Maudsley and Cambridge and Peterborough, building on previous work using anonymised data sets.

We reviewed data collected by the Royal College of Psychiatrists regarding numbers of older adult psychiatric posts and training rates to older adult posts across the country. Various existing data was compiled to show past demographic changes and future expectations in training.

**Results:**

The Older Adult UK cohort is growing, with a large cohort currently aged 55–65 soon to enter the over 65 age band. This group is projected to show increased complex psychiatric comorbidity which Older Adults services are poorly equipped to address. This will be an additional complication to the already complex medicalcomorbidity expected in Older Adult populations. This pattern of increasing psychiatric and medical comorbidity is only set to increase as prevalence of psychiatric disorders rises in the UK population.

Meanwhile, consultant provision in Old Age psychiatry is currently insufficient. RCPsych data demonstrates that 28.8% of Old Age psychiatry consultant posts in the UK are currently vacant or filled by locum doctors. Adjusted for population, the number of old age psychiatry consultants in England has been trending downwards over the last decade, with similar trends in the devolved nations. This is despite previous workforce projections from the Royal College which anticipate that the number of Old Age psychiatry consultant posts needs to double. Our collected data shows retirement rates have remained relatively static, implicating the major contributor to the current workforce shortfall is the consistent underfilling of Old Age psychiatry specialty training posts.

Our review of RCPsych census data shows that Older Adult training posts have been consistently underfilled across the country. The average fill rate across the last decade for Older Adult posts sits at 49% nationally with high inter-regional differences, contributing to over 300 posts not being filled across the decade.

**Conclusion:**

Without corrective action, Old Age psychiatry services are at risk of deteriorating,becoming increasingly unsafe and unable to accommodate the vulnerable populations they serve.

To address this the Older Adults Faculty at the Royal College is working to highlight these current and projected shortfalls and review the contributing factors to develop a new workforce plan.

